# Data Analysis Strategies for Protein Microarrays

**DOI:** 10.3390/microarrays1020064

**Published:** 2012-08-06

**Authors:** Paula Díez, Noelia Dasilva, María González-González, Sergio Matarraz, Juan Casado-Vela, Alberto Orfao, Manuel Fuentes

**Affiliations:** 1 Centro de Investigación del Cáncer/IBMCC (USAL/CSIC), IBSAL, Departamento de Medicina and Servicio General de Citometría, University of Salamanca, Salamanca 37007, Spain; Email: pauladg@usal.es (P.D.); noeliadf@usal.es (N.D.); mariagg@usal.es (M.G.-G.); smats@usal.es (S.M.); orfao@usal.es (A.O.); 2 Translational Oncology Unit, Instituto de Investigaciones Biomédicas ‘Alberto Sols’, Spanish National Research Council (CSIC-UAM), 28029 Madrid, Spain; Email: jcasado@iib.uam.es

**Keywords:** microarray, proteome, biomarker, algorithm, normalization, fluorescence intensity, background correction

## Abstract

Microarrays constitute a new platform which allows the discovery and characterization of proteins. According to different features, such as content, surface or detection system, there are many types of protein microarrays which can be applied for the identification of disease biomarkers and the characterization of protein expression patterns. However, the analysis and interpretation of the amount of information generated by microarrays remain a challenge. Further data analysis strategies are essential to obtain representative and reproducible results. Therefore, the experimental design is key, since the number of samples and dyes, among others aspects, would define the appropriate analysis method to be used. In this sense, several algorithms have been proposed so far to overcome analytical difficulties derived from fluorescence overlapping and/or background noise. Each kind of microarray is developed to fulfill a specific purpose. Therefore, the selection of appropriate analytical and data analysis strategies is crucial to achieve successful biological conclusions. In the present review, we focus on current algorithms and main strategies for data interpretation.

## 1. Introduction

The human proteome comprises ~23,000 protein-coding genes leading to >100,000 protein species mainly derived after alternative splicing and post-translational modifications (over a thousand post-translational modifications are currently listed in public databases). Thus, the overwhelming size and complexity of human proteome requires the development of high-throughput techniques enabling the detection of multiple proteins in a single analysis. Despite recent advances in proteomics technologies, only a small portion of human proteomes has been unraveled at the biochemical level. After the age of genomics, proteomics might seem to be a promising start to look deeper into the mechanisms of disease progression by generating individual protein expression profiles for every patient [1]. Consequently, proteomics may bring personalized medicine closer by implementing the five “Rs” criteria: right patient/target, right diagnosis, right treatment, right drug/target and right dose/time [2]. Therefore, the field of protein microarrays has expanded during the last decade, mainly due to the possibility of analyzing hundreds to thousands of proteins in a single experiment and in a high-throughput format [3]. Furthermore, high-density protein microarrays constitute a novel analytical tool that potentially will allow for biomarker discovery. The development and standardization of protein microarrays and automated data analysis of protein expression profiles might translate into a more accurate diagnostic and prognostic stratification of patients in clinical routine [4]. Despite the promising perspectives, the availability of multiple types of protein microarrays and the lack of standardized data analysis algorithms still hamper the widespread use of this technology. Here we provide an overview of the different types of protein microarrays and their application in order to address the human proteome and main data analysis methods currently available. 

## 2. Concept of Protein Microarrays and Current Applications

The idea of using microspots of antibodies printed on solid supports to develop more sensitive and quantitative assays was initially proposed by Roger Ekins in the late 1980s [5]. However, the project began to take shape in the late 1990s, with the introduction of DNA microarray technology. Indeed, Alejandro Zaffaroni and colleagues designed the first high-density microarray of peptides and oligonucleotides through photolithographic methods [6]. Briefly, protein microarrays are defined as miniaturized 2D arrays [7], which allow performing simultaneously high throughput studies of thousands of proteins [8,9,10] and subsequently the analysis of thousands of parameters within a single experiment. Moreover, it is also possible to compare two different samples labeled with two different fluorochromes on a single microarray [9,11,12]. In addition, this technology has been fruitfully employed in the identification, quantification and functional analysis of proteins in basic and applied research of proteomes [13,14], for example in antibody profiling or enzymatic studies [8,15,16].

During the last few years protein microarrays have provided the possibility to study protein-protein interactions and, to identify several biomarkers for different diseases [8,9] including neoplastic or autoimmune diseases, as well as to characterize targets for therapy protocols [9]. Indeed biomarker validation studies have been performed by protein microarrays. Certainly, several companies developed different microarrays formats, such as bead-based and planar microarrays [1,9,11,17]. It is noteworthy to highlight that microarrays have changed and implemented pharmaceutical research with continuously increasing diagnostic applications underlying multi-parametric measurements. In this sense, an additional advantage to be considered is the small amount of sample required for a single microarray, which is particularly favorable for patient management and, obviously when the sample amount is a limitation. On the other hand protein microarrays might be particularly relevant in disease monitoring and evaluation of minimal residual disease in the near future [11,12]. It is well known that a direct correlation between gene expression and protein abundance cannot be systematically established owing to post-translational protein modifications (e.g., glycosylation, phosphorylation and acetylation) [18]. For this reason, high-density protein microarrays remain necessary as they offer multiple improvements over conventional techniques, including better resolution, selectivity and sensitivity [19,20].

### 2.1. Types of Protein Microarrays

**Figure 1:**
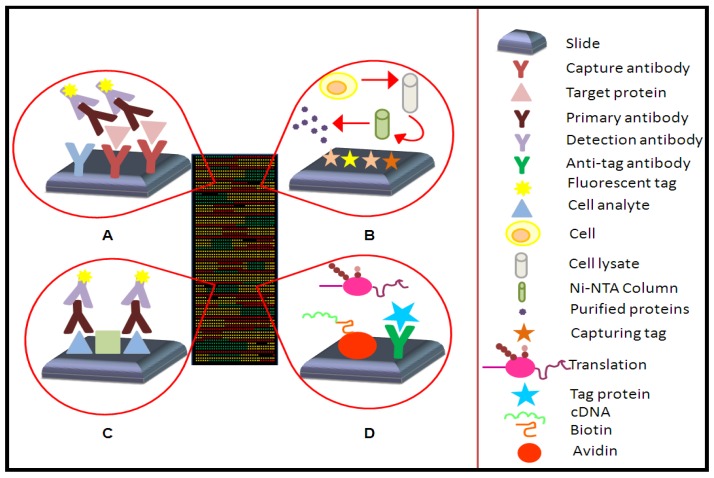
Types of different microarrays. (**a**) Capture arrays. (**b**) Cell-based protein microarrays. (**c**) Reverse phase arrays. (**d**) Cell-free nucleic acid programmable protein array.

According to their applications, the planar protein microarrays have been classified in three main categories: analytical microarrays, reverse phase arrays (RPA) and functional microarrays (Figure 1) [7,14,21]. On the other hand, microspheres bead based systems should also be considered, which use different size or color beads as a support of the capture agent to analyze the sample. In such microarray format, flow cytometry is coupled in order to support the identification of each specific binding according to the size, color and mean fluorescence intensity of conjugated fluorochromes [14].

#### 2.1.1. Analytical Microarrays

A range of capture agents, differing in composition, origin and, thus, differing in their affinity properties may be attached on microarrays [9]. Both, antigens or antibodies may be immobilized on the surface of slices and used as baits present in the test sample [7,14]. This kind of microarray is used to determine parameters such as the binding affinity and specificity and to study protein expression levels in complex mixtures [7,22], but also they cover clinical applications such as studies in immunology or biomarkers detection [9] and they can be used to monitor differential expression profiles, such as protein patterns in response to environmental stress or differences among a healthy tissue and with respect to a pathological sample [22]. In addition, analytical microarrays imply direct labeling protocols of thousands of proteins, which might be another critical limitation. The chemical labeling of proteins can destroy epitopes by covalent combination of dyes or haptens. Moreover, only selected target proteins can be analyzed by antibody microarrays [9,11,14].

#### 2.1.2. Reverse Phase Arrays

In this case, cellular or tissue lysate or even serum samples are immobilized on the microarray surface and the detection is completed through an antibody against the target proteins. To achieve a higher fluorescent signal to be detected, a secondary antibody conjugated with a fluorochrome is added to the first one. This ensures the signal intensity is directly related with the specificity, the binding affinity and the sterical accessibility of the antibody against the target protein [7,9,14]. The production of a functional map for cell signaling pathways from individual cells or tissues by RPA arrays has increased the interest on this kind of arrays with the objective of developing personalized therapies [7,9,23]. The proteins involved in RPA do not require labeling and only a little amount of protein is needed to produce the microarrays. However, fewer analytes can be analyzed due to the limited number of labeled antibodies for detection and also low availability of specific protein antibodies suitable for RPAs [7,10].

#### 2.1.3. Functional Microarrays

Functional microarrays are composed of full length functional proteins or protein domains and study the biochemical characteristics and functions of native proteins, as well as peptides or domains highly purified through cell-based methods or by cell-free expression on the microarray [9,11,21,22]. They allow studying the whole proteome in a single assay. Functional microarrays are also employed to examine the diverse protein interactions: protein-protein, protein-DNA, protein-RNA, protein-phospholipids and protein-small molecules [22]. 

*In situ* expressed microarrays, one of the subtypes of functional microarrays, are based on cell-free expression systems such as *Escherichia coli* 30 s, rabbit reticulocyte lysates (RRL) and wheat germ extracts [9], which have to be very well purified throughout chromatography or electrophoresis [7]. A library of open reading frames is also required [9,21,24]. 

Cell-free based protein microarrays have been applied to immunological studies [25], vaccine development [26,27], early detection of biomarkers [27,28], biochemical activity [21] protein-protein interaction studies [10,28], such as protein-protein, protein-DNA, protein-RNA, protein-phospholipids, and protein-small molecule interactions [22] and toxin detection [29,30]. Over the last few years, several *in situ *expressed microarrays have been developed such as: Protein *in situ* arrays (PISA), printing protein arrays from DNA (DAPA) arrays and Nucleic Acids Programmable Protein Arrays (NAPPA) [9,11,17].

NAPPA is one of the most relevant microarrays in this field. The DNA templates are bound onto the slide surface; the protein of interest is encoded and a GST tag is added. This is a fusion protein with a tag, which will allow binding to the slide. The biotinylated DNA plasmid is attached through an avidin to the aminopropyltriethoxysilane (APTES)-coated surface. In addition, RRL is used to carry out protein expression. There are also anti-GST antibodies attached to the slide, where the fusion protein joins. As a result, an array with the expressed protein and its corresponding DNA is achieved all on the same slide [8]. 

NAPPA is a good cost-effective technique because of the small volume of cell-free extract required for protein expression. Also, the use of immobilized DNA allows storage of the array for a long time until the next procedure. The main drawback is the invested time to generate the cDNA with the protein of interest and the tag, but even this method does not achieve a pure protein. On the other hand, high yields of high quality DNA were obtained for immobilization by using a diamine-derivatized resin. It was also found that BSA improved the binding efficiency of DNA and that is why a master mix of cDNA, antibody, BS^3^ and BSA is used [8].

### 2.2. Current Application of Protein Microarrays

Protein microarray technology has been successfully applied in different biomedical areas (Table 1).

**Table 1:** Current applications of protein microarrays.

Disease	Type of microarray	Object of study	Reference
Cancer	multiplexed array	CA-125; CA19-9; EGFR; C-protein; myoglobin; APOA1; APOC3; MIP-1; IL6; IL18; tenascin-C	Amonkar *et al. *2009
	NAPPA	p53	Dasilva *et al. *2012
Nodular thyroid disease	protein array	EGF; HGF; IL5; IL8; RANTES	Linkov *et al. *2008
	multiplexed array	cytokines; growth factors; cell adhesion molecules	Xiaobo *et al.* 2010
	reverse phase array	*Salmonella typhimurium*	Cid *et al. *2011
Infectious disease	antigen microarray	*Vaccinia* virus; *Yersinia pestis*	Natesan *et al. *2010
	antibody array	cholera; diphtheria; staphylococcal enterotoxin B; tetanus toxin; anthrax protective antigen	Rucker *et al.* 2005
	protein array	B lymphocyte	Wadia *et al.* 2011; Belov *et al. *2001
Systematic rheumatic disease	antibody microarray	nuclear proteins; nucleoprotein complexes	Dolores *et al.* 2001
Diabetes (type I)	NAPPA		Sibani *et al.* 2011

#### 2.2.1. Cancer

One of the most relevant applications of microarrays is the detection of biomarkers for many different diseases, including cancer, where the importance of an early detection is fundamental [11]. One example is the use NAPPA to address the detection of p53 auto-antibodies present in sera from breast cancer patients. Since the occurrence of p53 auto-antibodies is directly related to the tumor burden, detection of such auto-antibodies may lead to the recommendation of neo-adjuvant chemotherapy [30].

Also, capture microarrays have been used by Sreekumar *et al*. to monitor changes in protein abundance in colon carcinoma cells following exposure to ionizing radiation [31,32]. Amonkar *et al*. identified an 11-plex protein panel including: CA-125, CA 19-9, soluble epidermal growth factor receptor (EGFR), C-reactive protein, myoglobin, apolipoprotein A1 (APOA1), APOC3, macrophage inhibitory protein 1 (MIP-1), interleukin-6 and 18 (IL6 and IL18), and tenascin C. These authors built microarrays bearing this panel of proteins to analyze plasma samples and discussed their applicability to distinguish patients with ovarian cancer from controls [32]. 

On the other hand, over the last few years it has given relevance to the activity of protein kinases mainly because of its decisive function in the cell and the generation of specific treatment against them. With the growing knowledge generated about cancer biology, the new generation target drugs have different action mechanisms being more specific and causing less secondary damages for the patient. However, they have brought several challenges such as biomarker identification and developing technology to assure the drug carries out the desired function. Once these difficulties have been overcome, it is necessary to establish whether the patient outcome could be defined in function of kinase expression profile or based on the treatment. Eventually, patient monitoring determines the most accurate treatment to achieve the best prognostic. Also, it is necessary to identify the correct treatment responsive population for the precise therapy and avoid non-responsive patient groups. This was achieved for inhibitors of ERK/MAPK pathway, but a great effort still needs to be made to reveal other successful therapies [33]. 

#### 2.2.2. Immunology

Protein microarrays are also a valuable tool for the development of vaccines [11]. As an example of this application, Belov *et al*. developed an array of the expression of ninety different clusters of differentiation (CD) antigens from different leukemia cells. The slide was incubated with a cellular suspension and the cells with the matching CD bound to the corresponding antibody. As a result, they achieved different cell patterns when a control sera or a pathological sample was used, so each leukemia patient can be specifically characterized [14,34,35]. 

Systematic rheumatic disease, an autoimmune disease, can be accurately diagnosed thanks to the presence of nuclear proteins and nucleoprotein complexes which can be used as targets for antibodies in order to detect the phase of the disease [20]. 

NAPPA represents a good alternative to detect biomarkers in autoimmune diseases, such as systemic rheumatic disease, type I diabetes or systemic lupus among others, as has been demonstrated in different studies [36].

#### 2.2.3. Nodular Thyroid Disease

Nodular thyroid disease also can be analyzed thanks to protein arrays. Linkov *et al*. have defined patterns of serum/plasma biomarkers. In this way, epithelial growth factor (EGF), hepatocyte growth factor (HGF), IL5, IL8 and C-C motif chemokine 5 (CCL5, also known as RANTES) can be used as protein biomarkers of disease states [37]. Also, thyroid function can be analyzed, along with other analytes, such as cytokines, growth factors and cell adhesion molecules, on the same surface, with a microarray system which uses chemiluminescence as a readout signal.

#### 2.2.4. Infectious Disease

Understanding how pathogens are capable of modifying cell pathways can be a good strategy in order to establish preventive and therapeutic interventions for infectious diseases. Although there are not many relevant studies, RPA seems to be a good choice. In this way, Cid *et al*. studied the potential of RPA technology through cell cultures infected by *Salmonella typhimurium *[38]. In these kind of arrays, cell lysates are printed to a solid support so as to be analyzed by quantitative immunodetection. The base of the analysis consists of detecting the presence of proteins or their post-translational modifications in cell lines after exposure to pathogens under different conditions. Specifically, Cid *et al*. studied T3SS effectors in infected HeLa cells and have demonstrated that RPA allow quantifying the relative amounts of each marker protein in the samples [38].

Natesan *et al*. developed an antigen microarray to establish a signature of pathogen proteins displayed by *Vaccinia* virus and *Yersinia pestis*. The proteome microarray was adapted from a functional microarray in which genomic DNA of the vaccine strain was used as the template for PCR amplification. Then, recombinant viral proteins were generated as GST-tagged fusions. Later, GST-tagged proteins were purified to ≥90% homogeneity using affinity chromatography. Both, viral and control proteins were printed onto glass slides coated with a thin layer of nitrocellulose. Nearly 95% of the viral proteome were successfully expressed, purified and arrayed [18].

## 3. Feature Aspects of Protein Microarrays

Three main features of protein microarrays need to be considered during their design, such as type of surface used, molecules bound onto the surface and detection techniques selected. Depending on the type of array, the characteristics cited above can vary. For example, a protein microarray is not the same as an aptamer array. Thus, it is necessary to take into account the different designs when an array is developed, since the results may be contrary to all expectations, simply by choosing an inappropriate surface or detection format. The reasons specified above justify the need for careful selection of a number of features, as detailed in this section.

### 3.1. Array Capture Agents

Regarding planar arrays, capture agents are immobilized in rows and columns creating a set of spots onto the microarray ready to be exposed to the test sample. The binding between the capture agent and the analyte can be detected by label or label-free detection methods such as fluorescence, chemiluminescence, mass spectrometry, radioactivity or electrochemistry [9,11,12].

Although there are several different capture agents, antibodies are the most common. However, new approximations are being developed, e.g. phage display or mRNA display are the most promising techniques, but also, highly specific oligonucleotides and aptamers (Table 2) [12]. Employing monoclonal antibodies in routine array production incurs excessive costs due to the hybridoma technology and their production. Therefore, phage display is an appropriate substitute according to the easier and cheaper methods to obtain and purify the antibody fragments (Table 2) [23,39,40].

**Table 2:** List of capture agents used in protein arrays, source and technique.

Capture agent	Source of proteins	Technique
Mab *	mouse	Hybridoma
sc Fv */Fab * diabodies	antibody libraries	Phage display, *in vitr*o evolution
Affinity binding agents	recombinant fibronectin structures	*In vitro* evolution
Affibodies		
Aptamers (DNA, RNA, peptide)		
Receptors ligands	synthetic	Combinatorial chemistry
Substrates of enzymes	synthetic; pro-and eukaryotic organisms	Protein purification, recombinant protein technology(bacterial, fusion proteins, baculovirus, peptide synthesis)

* Abbreviations: Fab, antigen-binding fragment; sc Fv, single-chain variable region fragment; Mab, monoclonal antibody.

On the other hand, highly purified recombinant proteins are needed to satisfy the huge demand for capture agents. Because of that, new high-throughput techniques have to be developed, as well as, improving the existence ones. Moreover, these specific proteins allow generating microarrays which are analyzed faster, with high affinity bindings, permitting more efficient screening and also, avoiding or, at least decreasing, the cross-reactivity [12,17].

### 3.2. Array Surfaces

The slides used to immobilize the capture agent are usually made of glass, but can also be made of plastic, metal or polymer membranes [12,41] Since microarrays were first developed, surfaces have evolved from polyvinylidene fluoride (PVDF) membranes to glass slides. The most significant challenge is obtaining a surface which can take a variety of protein structures and compositions while preserving the function, structure and binding of each single protein. Immobilization is important, but also the capability of accurately detecting the protein-protein interaction. That is why three types of surfaces have been developed, each with a specific immobilization protocol. The first category is two-dimensional (2D) plain glass slides, which are activated with a diversity of coupling chemistries such as aldehyde, epoxy or carboxylic esters and they bind proteins or antibodies through electrostatic interactions or the generation of covalent bonds. The strength of the binding and the low variation enables rapid evaporation of the liquid environment and it is suspected that this affects the three-dimensional structure due to the close protein surface contact. On the other hand, there are three-dimensional (3D) gels or membrane-coated surfaces, for instance, polyacrylamide, agarose and nitrocellulose. They bind the protein by adsorption and seem to better safeguard the initial conformation; nevertheless, they present variations in signal intensity. Finally, the third type is a mixture of the previous ones: showing a supra-molecular structure at the surface which is why it is not a 2D slide, nor has it a visible 3D structure [39]. 

The immobilization of proteins is usually completed using non-covalent protein surface interactions with hydrophobic (e.g., nitrocellulose and polystyrene) or positively charged (e.g., poly-lysine and aminosilane) surfaces [12]. This kind of method attachment determines a random orientation of the proteins onto the slide because of the passive adsorption [22]. Covalent attachment achieves employing chemically activated surfaces (e.g., aldehyde, epoxy or ester functional groups), also the attachment is achieved by specific bimolecular interactions (e.g., streptavidin-biotin, His-tag-nickel-chelates) [12]. The uniform orientation of the different proteins onto the chip surface can be achieved using nickel coated slides for the His-tag use or Streptavidin slides [22]. The tiny microspots on the slide surface are made using contact printing arrayers with tiny needles placing sub-nanoliter sample volumes directly on the surface. However, non-contact deposition technologies are used that apply capillaries or ink-jet technology to deposit nanoliter-picoliter droplets onto the surface [12]. Immobilizing a protein onto a slide necessitates preservation of the conformation and the function of the protein, as well as, the binding capacity [22].

Finally, it is worth mentioning that thanks to flow cytometry, a new type of array has been developed, called the color-coded microsphere array. Each microsphere is covered with different antibodies, and each antibody can be detected through different fluorochromes; thereby, monitoring the binding antibody-antigen. The latter is attained from the sample of interest, can be fast, accurate, low cost and highly-sensitive. A new system of magnetic microspheres has been designed recently with high-reproducibility and sensibility, low background noise and price, and having a wide dynamic range [9,11].

In summary, a color-coded suspension microsphere array presents the following advantages over current antibody arrays: (*i*) High level of multiplexing. (*ii*) A sensitive, accurate and wide dynamic range signal readout. (*iii*) Information about protein-protein interactions. (*iv*) Flexibility in array composition. (*v*) Automatic preprocessing (gating, QC) in a short time. (*vi*) Clinical applications. All due to the integration of flow cytometry, antibody array detection of size-resolved lysates and computer-assisted data processing [42].

### 3.3. Array Detection Technologies

Many different detection techniques were developed over time that enable reliable, sensitive, and specific detection of arrays in a high-throughput manner. Some of them are based label tools and others are label-free techniques. The first type involved a fluorochrome or radioisotope tag molecule for the query element [7,9,11,39] or some new tags recently have been developed, such as quantum dots (QDs), gold nanoparticles (NPs), Raman-dye label carbon nanotubes or silica NPs [7,11]. However, these techniques can interfere with the probe’s capacity to bind to the target protein [22]. To solve these problems, label-free techniques have been developed. They include surface plasmon resonance (SPR), carbon nanotubes, microelectromechanical cantilevers and surface-enhanced laser desorption ionization (SELDI)-TOF-MS. These techniques can measure the mass, dielectric or optical properties of the query molecule [9,11,22]. Currently, only SPR is a label-free technique generally available in many laboratories. Although the other tools need to be developed to become suitable high-throughput techniques, they show huge potential in protein microarrays [9].

**Figure 2:**
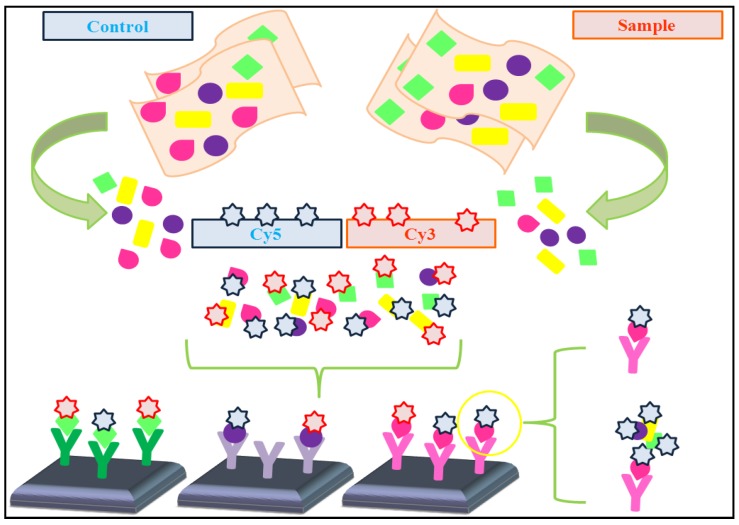
Microarrays for differential protein displays. Proteins from controls and samples are isolated and conjugated to different fluorescent molecules, for example Cy3 and Cy5. The samples are mixed in equal amounts and incubated simultaneously on an antibody microarray. Then, target molecules will be captured by their specific antibody and the differences in protein expression are directly reflected by the overlay of the color signal. The section marked with a yellow circle reflects the difficulty of quantification because a prominent signal could be the result of a single protein, but also of a large protein complex.

The fluorochrome labeling technique, directly incorporated from the DNA-chip technology, has become widely used due to its effectiveness and its compatibility with different systems of laser scanning [9,22]. Both, antibodies and antigens are labeled with two different fluorochromes, mixed and concurrently incubated on the same array. Then, dual color detection systems allow the detection of both signals and measure their ratios. Finally, the analysis of data reveals whether the targets are present in different or similar concentrations on each spot (Figure 2) [12,14] and allowing differentiate between two separate samples [12,39]. The fluorochrome is bound to the antibody or to antigen depending on the type of array [12,14]. A key advantage is the possibility of comparing two samples without the need of a second independent array, since both assays are performed on the same slide [39].

Achieving a more powerful signal is possible using indirect labeling, which is the most common system with serum samples, antibodies and in sandwich assays. However, cross-reactivity can be caused; hence the assortment of antibodies able to be employed is limited [39]. Anyway, these problems can be solved using different strategies, such as labeling procedures based on enzymatic signal amplification. In this way, Schweitzer *et al*. developed a method which requires enzymatic extension. This can be done through rolling circle amplification (RCA) which is based on the enzymatic extension of a primer-antibody conjugate followed by hybridization of labeled probes [43]. Also, tyramide signal amplification (TSA) system can be utilized [44]. On the other hand, Huang *et al*. have employed chemiluminescence in order to achieve the required purpose [45]. However, sometimes, these strategies are not sufficient to solve the cross-reactivity problems. When these problems appear, together with others, data analysis is the tool which allows obtaining correct conclusions from experiments, by taking the problems derived from the hybridization method into account and solving them. However, there are no simple and uniform strategies for the analysis of data obtained by different kinds of protein microarrays. Thus, the development of user-friendly and accurate algorithms still needs to be developed. 

## 4. Data Analysis Methods

**Figure 3:**
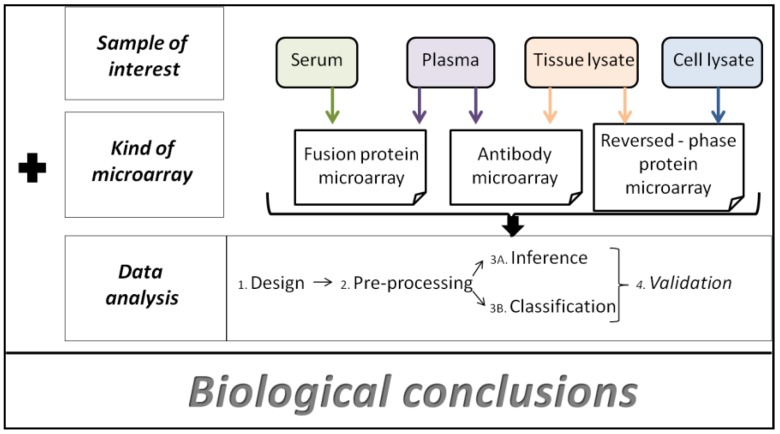
Workflow of protein microarray development in which the sample of interest, the type of microarray and the data analysis strategy are essential for biological conclusions.

The wealth of information generated by protein microarrays may provide solid evidence concerning protein functions, their interactions and even their involvement in signaling pathways. Interestingly, these data can also be applicable as a tool for clinical diagnostics. Nevertheless, the translation of data into meaningful information requires automated data processing and handling. As depicted in Figure 3, data processing and analysis is inherent to protein arrays. Thus, it becomes a crucial step in the search for solid biological conclusions [46]. There are several strategies to analyze protein data, some of which have their origin in DNA microarray analysis, such as spot-finding on slide images, Z-score calculations and significance analysis of microarrays (SAM). However, concentration dependent analysis (CDA) has been specifically developed for protein microarrays [46]. 

• Spot intensity determination: microarray image analysis starts with the fixing of spot intensity. Generally, for this task, GenePix Pro software (Molecular Devices, Union City, CA) is used. First of all, a grid of circles must be placed over the protein spots. Their position and size have to be adjusted in order to get reliable intensity data. Finally, an output file is created by the program.
• Z-score analysis: the Z-score equation, 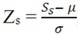, where Z_s_ is the Z-score for the *s*^th^ spot, S_s_ is the signal for that spot, µ is the mean signal across all spots and σ is the standard deviation across all spots, is an interesting tool to determine which signals are significantly different from the expected value and which are not.
• Concentration-dependent analysis (CDA): due to the quantity of spotted proteins on the slide, absolute signals are affected by protein concentration. As a means to solve this issue, a different Z-score, 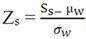, can be calculated to remove outliers. This novel Z-score is calculated using an iteration process that is repeated until every spot signal measured is in accordance with the mean value. In the equation above, Z_s_ is the Z-score for the *s*^th^ spot, S_s_ is the signal for that spot, µ_w _is the mean signal for the spots within the window and σ_w_ is the standard deviation for spots within the window.

### 4.1. Dual-Color *vs.* Single-Color Assays

Antibodies immobilized on the microarray surface can be detected through direct labeling, requiring only a single capture antibody specific for each target protein. Alternatively, a sandwich approach can be carried out, which consists of two sets of antibodies, the first one is specific for the target protein, and the second one for the first antibody [47]. Then, the signal is detected by a colorimetric reaction or a fluorescent dye. This last alternative enables a dual-color layout that is based on labeling each sample with different fluorescent dyes (e.g., Cy3 and Cy5), which competes for the binding sites of the antibodies immobilized on the array. After the incubation, intensity signals are measured for each dye using fluorescence image scanners. Dual-color assays typically display better reproducibility and discriminative power compared to single-color assays [47].

* Single-color assays: Olle *et al. *[47] developed a single antibody-based microarray which presents standard antigen concentration. Also, it uses an internal controlled system based on two colors, one for the amount of antibody spotted and the other for the amount of the antigen used for the quantification of the level of protein expression. To validate this microarray, levels of protein expression were compared with results obtained by western blot analysis and the data were similar, although the sensitivity was higher with the microarray. In their study, they show that this microarray has not only the potential to accurately assess proteins in complex fluids, but also a large range of linearity.
* Dual-color assays: Data pre-processing protocols are usually applied to prevent undesired technical artifacts. These protocols frequently include the following steps (adapted from [48]):
  • Filtering, in order to remove failed and low-quality spots.
  • Background correction, to avoid fluorescence signal due to non-specific binding.
  • Data normalization, aimed to reduce variations between the two samples co-hybridized on each array and also between arrays.

All the steps mentioned above are commonly used in protein arrays due to the difficulty of quantifying protein expression in a multiplexed manner. Multiple causes may lead to the occurrence of such artifacts, including the effect of electric charges, hydrophobic interaction of proteins, artifacts due to differences in protein sizes and antibody/antigen binding kinetics. For all these reasons, microarray data frequently require normalization. Several methods can be used for data normalization, such as: (*i*) house-keeping probes, (*ii*) inclusion of spike-in controls and (*iii*) use of algorithms to define sets of probes. In the following, these methods are described [48].

Different microarray designs may be considered (Table 3, adapted from [48]) which differ in the number of samples and type of array employed, as described below:

• Reference design: the sample of interest is labeled with one fluorescent fluorochrome (e.g., Cy3), whereas the single reference sample is labeled with a different fluorescent fluorochrome (e.g., Cy5). In this type of design, it is necessary to calculate the log ratio of dyes intensities.
• Balanced-block design: two samples which are hybridized, bearing two different fluorochromes (Cy3 and Cy5). Then, samples are balanced with respect to dyes. In this case, the microarray is considered as a block.
• Incomplete-block design: more than two samples are co-hybridized on the microarray, whereas only two fluorochromes are used (Cy3 and Cy5). Despite this, samples are balanced.
• Loop design: each sample is hybridized in a different array using a different fluorochrome. This supposes a great disadvantage because the number of arrays is duplicated.

Balanced-block and loop designs allow correct dye effect normalization, which are required for the correct analysis of two-dye systems. The first one is used for comparison studies, whereas the loop design is useful for discovery studies [48]. The table below shows the relations between samples and dyes used in the experimental designs indicated above [49]. 

**Table 3:** Types of microarray experiment designs using two colors. A_i_: sample *i* from class A; B_i_: sample *i* from class B; C_i_: sample *i* from class C; R: reference sample. In reference design, A_i_ and B_i_ are labeled with Cy5, whereas R is labeled with Cy3. In the rest of the designs proposed, each class is labeled with a dye, typically Cy5 and Cy3 [49].

EXPERIMENTAL DESIGN	ARRAY #1	ARRAY #2	ARRAY #3	ARRAY #4
Reference	A_1_/R	A_2_/R	B_1_/R	B_2_/R
Balance block	A_1_/B_1_	B_2_/A_2_		
Incomplete block	A_1_/B_1_	B_2_/C1	C_2_/A_2_	
Loop	A_1_/B_1_	B_1_/A2	A_2_/B_2_	B_2_/A_1_

#### 4.1.1. Rank-Invariant Selection Algorithm (InvTseng)

This algorithm is especially valuable in those cases where house-keeping controls are not available. Tseng *et al. *[50] suggested a strategy which enables selecting a set of non-differentially expressed proteins. This method, applied to dual-color arrays, is an adaptation of the invariant difference selection algorithm (IDS) used with single-channel microarrays.

A protein *p *is considered to be rank-invariant on an array, if the difference of the ranked Cy5 and Cy3 intensities is less than a threshold *d* and the average of the ranked intensities is not among the highest or lowest *l *ranks. For each array *j*, the set of rank-invariant proteins 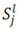 is determined by the following expression [50]:


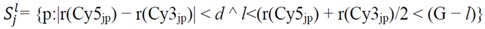


where r(Cy5_jp_) and r(Cy3_jp_) are the ranks of the intensities and *G *is the number of spotted proteins.

It has to be noted that a major limitation of the InvTseng algorithm proposed by Tseng *et al. *[50] is that it does not cover the entire intensity range.

#### 4.1.2. Modified Rank-Invariant Selection Algorithm (In-vMod)

The intensity range limitation mentioned above can be partially solved using the modified rank-invariant selection algorithm (In-vMod) [50] depicted as follows:


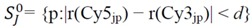


The In-vMod algorithm corrects the intensity values through the extrapolation of the curve to the lower and upper intensity limits. 

#### 4.1.3. Rank Difference Weighted Global Loess (RDWGL)

This next algorithm is applied to the whole probes on the array (w_jg_) to get a global normalization [50].


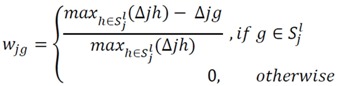


where Δ*_ig_* = |r(Cy5*_jg_*) − r(Cy3*_jg_*)| is the absolute difference of the ranked intensities of protein *g* on array *j. max* refers to the maximum value.

On the other hand, several standard normalization methods are used in order to correct the background of the slides including: (*i*) global loess normalization (GL); (*ii*) variance stabilizing normalization (VSN) and (*iii*) generalized procrustes analysis (GPA). GL fits a non-linear loess curve in which equal weight is assigned to all probes. VSN is based on the stabilizing of the variance of the transformed intensities to be approximately independent of the mean intensities. Finally, GPA scales and aligns matrices with the same dimension as a mean to normalize data [50].

### 4.2. Automated Analysis of Highly Complex Flow Cytometry

Stuchlý *et al. *developed a protein-profiling tool which allows computational feasibility, hands-on time, standardization and reproducibility, quality control feedback loops, data normalization and presentation of the results in an appropriate way to be analyzed [42]. 

Size exclusion chromatography-resolved microsphere-based affinity proteomics (Size-MAP) is a new tool that permits obtaining information about protein sizes, protein complexes and protein profile changes through flow cytometry detection. The statistical method used was based on modifications of Partitioning Around Medoids (PAM). Previously, they have tried to resolve individual color-coded microsphere types by using k-means clustering, model-based clustering, minimum spanning tree clustering, and hierarchical clustering. But, finally, PAM was seen as the best method. In this way, they adopted the standard approach of sequential two-dimensional gating. Kernel density was used to analyze the distribution of fluorescent signal [42].

• Automated gating of color-coded microspheres: the automated tool is responsible for the specific identification and differentiation among microsphere types and the consequent allocation of the code to each one.
• Analysis of size-MAP data: quantification of antibody-bound proteins amounts was determined with the medians of the fluorescence label signals. Next, these data will be processed through quality control (QC), normalization and analysis.
  ○ Quality control (*QC*)*:* first of all, the number of microspheres of each population is checked. Next, the density function of the signal is also determined.
  ○ Normalization: it is necessary to remove background noise and to establish protein sample differences. With the purpose of correcting the noise, the signal, from empty microspheres (those without any antibody), is subtracted from the signal of the microsphere population of interest.
  ○ Analysis: each protein entity has to be established and, for this purpose, fractions constituting specific protein entities must be defined. Then, signals for each fraction are summed up, representing the final result, which is the relative amount of a particular protein entity.

### 4.3. Data Analysis Methods from cDNA Arrays

Initially, in order to analyze antibody microarrays, strategies from cDNA arrays were implemented. Fluorescent dyes (e.g., Cy3 and Cy5) were used to label each sample and, then, log ratio of the intensities was calculated for every feature on the array. Nevertheless, this design was not the optimal one [48]. Therefore, two new methods were developed: balanced-block design and loop design. The first technique considers the arrays as a block, in such a way that samples hybridized are balanced with respect to dyes. In the second strategy, each sample is hybridized onto two different arrays, each with a different dye. Both designs allow suitable dye normalization. However, using two arrays for each sample supposes a considerable disadvantage [48].

The normalization is based on an internally normalized ratio (INR). A reference sample is labeled with fluorescent dye (e.g., Cy5), whereas the sample of interest is labeled with another dye (e.g., Cy3). In other arrays, the same samples are labeled with the opposite dyes (Cy5 for the sample of interest and Cy3 for the reference sample). Then, the ratio of both fluorescent dyes is calculated for each array. The INR is the geometric mean of the two ratios [48]. Also, ANOVA models, mixed ANOVA models and within-print tip local regression smoothing methods have been developed for the removal of systemic effects typically found in cDNA arrays [48].

### 4.4. Reverse Phase Array Data Analysis

RPA analysis is based on the construction of a serial dilution curve, which is characterized by two main advantages [51]: first, the signals in successive dilutions can be related to each other. In this way, protein concentration and signal intensity can be accurately established. Second, data quality can be checked due to raw data display. In RPA analysis several steps must be carried out, as follows:

• Serial dilution curve: the monotonic s-shaped response curve is described by Sips model:

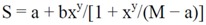


In the algorithm displayed above, *a* corresponds to the background noise; *b *is the response rate in the linear range; *M* is the maximum or saturation level and *x* is the concentration of the protein. Also, the equation can be modified in order to avoid data about protein concentration. In this way, *y* can vary. Generally, *y*≠ 1 applies to condition in which there is some heterogeneity in the solute molecules or the surface receptors. *y *approaches to 1 when the range of the free energy of binding shrinks to a singular point. In this last case, the equation is equivalent to the conventional Langmuir model.

• Parameterization of the serial dilution curve: a non-linear regression model is used to find the optimal parameters.
• Estimating protein concentrations: first of all it is necessary to check if protein concentration is saturated. This occurs if the M/r ratio is lower than signals measured. Then, the minimum and maximum of *x *(x_min _and x_max_, respectively) are estimated with these formulas:

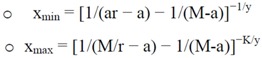


where *M/r* is a threshold value in which *M *is the saturation level and *r* should be >1. *K* refers to the *K*^th^ dilution step.

In summary, the Sips model presents physically meaningful parameters and has the optimal conditions for RPA experiments [51].

## 5. Conclusions

Microarray analysis includes four main steps which must be followed, such as design (surfaces, content, detection method), data preprocessing, inference, classification and validation. All these variables may significantly differ depending on the kind of microarray used. Therefore, it is important to bear in mind that different data analysis methods are also required [52]. 

**Array design** is crucial and may drastically affect data analysis. For that reason, careful design of microarrays is required, since it may significantly influence data analysis and final interpretation. It is recommendable (if not compulsory) that biological replicates are included in the microarrays, providing greater statistical confidence. Nevertheless, the introduction of replicates necessarily introduces more data to be analyzed, adding more complexity to the evaluation of the results [52]. 

**Data preprocessing** (*i.e.*, image analysis, normalization and data transformation) is the second step. Image analysis is made using image-processing algorithms that distinguish foreground from background intensities. To date, it is not known which method is the best for this purpose [52]. 

**Inference** is based on statistical strategies, which also incorporate variability in the analysis [52].

**Classification and validation: **Not only the large amount of data that are generated, but also the wide variety of results obtained which can be generated according to the type of array, are the reasons that explain why different data analyses are required. Since antibody microarrays and phage display arrays are different, the results obtained also differ [52]. This fact offers some advantages but also some disadvantages. On the one hand, the development of diverse types of arrays provides a variety of tools that enable disease analysis from multiple perspectives. Nevertheless, the main drawback is that there is a lack of standard analytical strategies, including array data processing. Briefly, a range of diseases are studied using different types of arrays, which are analyzed following different strategies. This introduces complexity in the analysis and the need of data analysis strategies based on different algorithms, image processing or validation methods [52].

Despite the differences among data processing methods applicable to microarray analysis, several general recommendations need to be considered [52] as follows: (a) using Bayesian approaches to examine intersections between sets of findings and evaluate multi-component hypotheses; (b) quality-control and validation methods are required; and (c) standardized testing platforms are needed.
